# Drug Facilitated Sexual Assault: Detection and Stability of Benzodiazepines in Spiked Drinks Using Gas Chromatography-Mass Spectrometry

**DOI:** 10.1371/journal.pone.0089031

**Published:** 2014-02-19

**Authors:** Lata Gautam, Sarah D. Sharratt, Michael D. Cole

**Affiliations:** Department of Life Sciences, Faculty of Science and Technology, Anglia Ruskin University, Cambridge, United Kingdom; National Institutes of Health, United States of America

## Abstract

Benzodiazepines are detected in a significant number of drug facilitated sexual assaults (DFSA). Whilst blood and urine from the victim are routinely analysed, due to the delay in reporting DFSA cases and the short half lives of most of these drugs in blood and urine, drug detection in such samples is problematic. Consideration of the drinks involved and analysis for drugs may start to address this. Here we have reconstructed the ‘spiking’ of three benzodiazepines (diazepam, flunitrazepam and temazepam) into five drinks, an alcopop (flavoured alcoholic drink), a beer, a white wine, a spirit, and a fruit based non-alcoholic drink (J2O) chosen as representative of those drinks commonly used by women in 16–24 year old age group. Using a validated GC-MS method for the simultaneous detection of these drugs in the drinks we have studied the storage stability of the benzodiazepines under two different storage conditions, uncontrolled room temperature and refrigerator (4°C) over a 25 day period. All drugs could be detected in all beverages over this time period. Diazepam was found to be stable in all of the beverages, except the J2O, under both storage conditions. Flunitrazepam and temazepam were found not to be stable but were detectable (97% loss of temazepam and 39% loss of flunitrazepam from J2O). The recommendations from this study are that there should be a policy change and that drinks thought to be involved in DFSA cases should be collected and analysed wherever possible to support other evidence types.

## Introduction

Drug-facilitated sexual assault (DFSA) is sexual activity (i) without the consent or (ii) the invalid consent of a participant when the victim is under the influence of drugs, noxious substances or chemical agents [1]. The use of sedatives for DFSA is not a new concept, in fact related legislation has existed in the United Kingdom since the Offences Against the Person Act, 1861 followed by the Sexual Offences Act, 1956 and 2003 [2]. DFSA has, in recent years, been classified as either ‘pro-active’ or ‘opportunistic’. Pro-active DFSA relates to the covert administration of a substance to the victim where purpose of the perpetrator is sexual assault. Opportunistic DFSA relates to cases where the victim has been rendered nearly or fully unconscious by their own actions prior to the sexual assault [1]–[3]. Previous literature indicates an increase in the number of claims of DFSA [3]–[4], which could be due to the advent of drugs which are easier to administer without the victim’s knowledge and better media coverage leading to increase in awareness [5]–[6].

Benzodiazepines are used by those who wish to commit DFSA because drugs such as diazepam, temazepam and flunitrazepam have a sedative effect and ability to cause amnesia [7]–[9]. These drugs are readily absorbed in the gastro-intestinal tract when taken orally with food which only delays the rate, but not the extent of absorption [10]. The absorption leads to altered mental state, drowsiness, confusion, slurred speech and lack of co-ordination [8].

Benzodiazepines have also been found to be present in a significant number of suspected DFSA cases [2], [10]–[15]. It should be noted that some of these positive samples may be due to prescribed or voluntary ingestion [2]–[3], [10] but the difference is not reported in the papers.

The three benzodiazepines were chosen because flunitrazepam is widely considered as date rape drug, diazepam is the most frequently encountered benzodiazepines in DFSA cases [11] and temazepam is a metabolite of diazepam and is also frequently encountered in casework [2], [16]. With regard to the link of flunitrazepam in DFSA cases, Roche Products Limited, manufacturer of Rohypnol (active ingredient flunitrazepam), has reformulated the drug by making its dissolution more difficult and generating a bright blue colour [17]. However, other preparations that contain flunitrazepam as their active substance (Flunipam^R^: 1 mg flunitrazepam, Alpharma Inc, Norway; Hypnodorm^R^: 1 mg flunitrazepam, Alphapharm Pty Ltd, Australia) are still available in the market.

Blood and urine are routinely collected for toxicological analysis. Due to the short detection window of benzodiazepines in blood (48 h) and urine (96 h), with some exceptions (12 h for midazolam, 7 days for diazepam) [8], alternative specimens such as hair may be preferred. Benzodiazepines are detectable in hair for months and years depending on the length of the sample. Research has been encouraged into such non-routine specimens as the time frame between the assault taking place and the reporting is often prolonged [11] negating the use of blood and urine samples [6], [15].

The testing of drink samples may also be of use in clarifying the events relating to alleged sexual assault where there has been a delay in reporting or collection of routine toxicology samples [18]. Empty glasses, bottles and vomit have all been submitted to the Forensic Science Services (FSS) in the UK for analysis in cases of alleged DFSA [2]. The conditions and duration of handling and storage of specimens for analysis have the potential to alter drug concentrations considerably. As initial concentrations of drug are generally of interest toxicologically, knowledge of the stability of these drugs in different matrices, and therefore the potential for variance between detected concentration and initial concentration, is important [19].

Benzodiazepines have been evaluated for stability in relation to storage temperatures for pre-hospital purposes - this included injectable solutions in clear glass syringes. In this sterile setting it was found that both diazepam and lorazepam showed significant decreases in concentration at ambient temperatures and at 37°C, and that refrigeration did not completely halt the process [20]. The stability of benzodiazepines under long term storage conditions in biological matrices has been researched with material relating to saliva, blood and urine samples. For whole blood samples storage at below −20°C has been recommended to minimise loss of concentration [21]. Similarly, another study reported that storage at ambient temperatures of bromazepam in plasma lead to degradation [22] and blood and plasma samples spiked with thirteen benzodiazepines and metabolites stored at 4°C showed at least 60% loss of analytes compared to original levels after 240 days [23]. This reflects the general recommendation that clinical or forensic specimens should be refrigerated, or preferably stored at −20°C or lower, to avoid degradation [19].

It is clear that there are a number of potential issues relating to the storage of benzodiazepine containing samples – the stability of the drugs themselves and the potential interactions with the matrix that they are contained in. A lack of research relating to the stability of benzodiazepines over time in different drinks and storage conditions has been identified.

A GC-MS method has been validated and applied to common beverages spiked with benzodiazepines to simulate casework samples stored under different controlled conditions at increasing time intervals. This research will inform law enforcement officers and analysts about the collection, storage conditions, duration of storage, analytical conditions and interpretation of data when drinks alleged to have been used for DFSA are analysed.

## Experimental

### Materials and Chemicals

All chemicals used were analytical reagent grade or better. Chloroform, ethyl acetate, isopropanol and methanol were purchased from Fisher Chemicals; eicosane was purchased from Acros Organics; diazepam, diazepam-d_5_, flunitrazepam and temazepam were purchased from Sigma Chemical Company; bis(trimethyl)trifluoroacetamide (BSTFA) containing 1% trimethylchlorosilane (TMCS) derivatising agent was purchased from Supelco Analytical.

The beverages used were Bacardi Breezer, an alcopop, orange flavour, 4% by volume, Becks Beer, 5% by volume, Hardy’s Classic Selection Chardonnay 2010, 12.5% by volume, J2O apple and mango, Chekov Imperial Vodka, 37.5% by volume, and were all purchased from local supermarket stores in Cambridge, UK during the period Oct 10–July 2011. The five drinks tested in this research were selected to represent common drink types consumed by women in the 16–24 years age group – a group at considerable risk of being the victim of DFSA.

### Standard Solutions

A mixed drug standard of diazepam, flunitrazepam and temazepam was prepared with each at a concentration of 100 µg/mL in methanol. When appropriate, the internal standard (IS), diazepam-d_5_, was added to give a final IS concentration of 25 µg/mL.

### Derivatisation of Samples

To derivatise the drugs prior to analysis owing to thermal instability of temazepam, each solution was evaporated to dryness using a miVac sample concentrator (Genevac Ltd., UK) at 30°C. Derivatisation was performed by addition of BSTFA: TCMS (40 µL, 99∶1%) and IS eicosane (20 µL, 100 µg/mL) in ethyl acetate and heating to 70°C for 15 minutes. The samples were analysed immediately after derivatisation.

### Instrumentation and Compound Identification

A Shimadzu QP 2010 GC-MS fitted with a ZB1 column (0.25 µm layer thickness, 0.25 mm i.d.) was utilised with split injection (14∶1) at 250°C. The injection volume was 1 µL. The column flow rate (He carrier gas) was 1.2 mL/min. The oven temperature programme was 150°C (2 min), then increasing at a rate of 20°C/min to 280°C and held at 280°C for 5 minutes. The quadrupole mass analyser was operated in electron impact (EI) mode. The ion source temperature was 230°C, interface temperature was 300°C and solvent delay time was 3 minutes. Samples were analysed in full scan and extracted ion modes which were used for data analysis. Ethyl acetate blanks were run between all samples, controls and standards to prevent carry over. Compounds were identified on the basis of retention index (compared to diazepam-d_5_) and mass spectrum. An unspiked sample of each of the beverages was prepared using the extraction and derivatisation method and was analysed in an identical manner to all other samples. This was to demonstrate that there were no drugs or compounds giving identical data in the reagents and drink matrices. In addition, linearity on the drug response was established using pure drug standards as well as spiked beverages.

### Sample Preparation: Drink Spiking and Storage

The Bacardi Breezer, Becks Beer, Hardys Chardonnay, Chekov Imperial Vodka and J2O (20 mL) were each spiked with individual drug solutions to give a final concentration of 10 µg/mL (diazepam and temazepam) and 20 µg/mL flunitrazepam. The difference in concentration is owing to the instrumental response. The beer and the Bacardi Breezer were degassed using an ultrasonic bath prior to addition of the drugs. Spiked beverages were divided into two aliquots (10 mL each) and stored at room temperature, which fluctuated diurnally, and in the refrigerator at 4°C, until liquid liquid extraction (LLE) and GC-MS analysis was carried out.

### Extraction of Benzodiazepines from Spiked Drinks

The IS (diazepam-d_5_) was added to individual drinks (0.5 mL aliquots), to give a final concentration of 25 µg/mL, immediately prior to extraction. LLE was performed using chloroform: isopropanol (1∶1 v/v, 100 µL×3) directly after spiking and on unspiked beverages [18]. Once extracted, the organic phase (bottom) was collected and subjected to analysis.

### Method Validation and Quantitation

Each of the beverages (10 mL) was spiked using individual drug solutions of 1 mg/mL diazepam and temazepam; and 2 mg/mL flunitrazepam to give the concentrations range of 2.5–50 µg/mL for diazepam and temazepam; 5–100 µg/mL for flunitrazepam. Analytes were extracted, derivatised and analysed as described above.

### Simulated Case

White wine samples were spiked with individual drugs to give final concentrations of 0.833 mg/mL each of diazepam and temazepam to simulate casework samples. This concentration was selected within the linear range. Each sample was analysed using the extraction and derivatisation method described above. Once the analysis was complete the identity of the drugs and the quantities detected were compared to those expected.

## Results and Discussion

### Compound Identification

The GC-MS method reported here was followed after derivatisation that allowed identification of temazepam, whereas diazepam and flunitrazepam could have been analysed without derivatisation. Sample injection without derivatisation of temazepam resulted in its decomposition. Temazepam is known to be thermally labile [24]. The data obtained for the standard drugs and drugs extracted from the drinks were identical with respect to retention index (Table 1) and mass spectrometry (Table 2). This allowed identification and quantification of the drugs. Solvent blanks and sample matrices were analysed to ensure there was no carryover between sample injections. Results from the matrix blank and negative controls demonstrated the absence of drugs in these samples. This proves that the chromatographic peaks and mass spectroscopic data could only have come from the drugs themselves and that there was nothing in any of the drinks tested which gave identical data indicating selectivity of the method.

**Table 1:** Compound identification parameters (n = 27).

Spiked drinks	Diazepam		Flunitrazepam		Silylated temazepam	
	RT^a^ ± RSD^b^	RI^c^	RT^a^ ± RSD^b^	RI^c^	RT^a^ ± RSD^b^	RI^c^
Pure drug	9.30±0.013	1.001	10.29±0.030	1.104	10.23±0.061	1.10
Bacardi Breezer	9.32±0.115	1.002	10.28±0.051	1.110	10.22±0.056	1.10
Becks Beer	9.32±0.041	1.001	10.28±0.050	1.110	10.22±0.054	1.10
Hardys Chardonnay	9.32±0.040	1.001	10.28±0.055	1.110	10.21±0.055	1.10
Chekov Imperial Vodka	9.32±0.042	1.001	10.28±0.048	1.110	10.22±0.046	1.10
J2O Apple & Mango	9.32±0.036	1.001	10.28±0.042	1.110	10.21±0.050	1.10

a Retention time/min.

b Relative standard deviation/%.

c retention index = RT of analyte/RT of Internal standard.

**Table 2:** Mass spectrometry data for diazepam, flunitrazepam and silylated temazepam as pure compounds and after extraction from beer as exemplar data.

Drug	Ion (m/z)	% intensity of pure compounds	% intensity after extraction from spiked beer
Diazepam	285	42	42
	284	74	71
	283	97	94
	257	46	43
	256	100	100
	221	33	36
Flunitrazepam	313	67	69
	312	100	100
	286	97	90
	266	61	59
	238	43	40
	183	20	20
Silylated temazepam	372	19	21
	345	36	39
	344	25	26
	343	100	100
	283	31	37
	257	36	44
	221	6	7

### Method Validation Results

Extracted ions (diazepam m/z = 256; diazepam-d_5_ m/z = 261; flunitrazepam m/z = 343 and temazepam m/z = 312) were utilised to construct calibration graphs for the pure drugs – the regression equations and r^2^ values are provided in Table 3. A linear detector response range was established for each drug in the ranges 0.025–0.25 mg/mL for diazepam and temazepam; and 0.05–1 mg/mL for flunitrazepam. The limit of detection (LOD) and limit of quantification (LOQ) were calculated using IUPAC (International Union of Pure and Applied Chemistry), 2009 [25] and are also given in Table 3. LOD ranged from 0.50–0.71 ng and LOQ ranged from 0.64–1.29 ng on column.

**Table 3:** Calibration and mass spectra interpretation for diazepam, silylated temazepam and flunitrazepam standards.

Drug	Regressionequation	R^2^ value	LOD^a^/ng	LOQ^b^/ng	Majorions/m/z	Interpretation	% abundance
Diazepam	Y = 27.7× +0.27	0.978	0.571	0.643	256	[M-CO]^+^ or[M-(H+HCN)]^+^	100
					283	[M-H]^+^	97
					221	[M-(CO+Cl)]^+^ or[M-(H+HCN+Cl)]^+^	33
Flunitrazepam	Y = 12.02×–0.797	0.983	0.714	1.286	312	[M-H]^+^	100
					286	[M-HCN]^+^	97
					266	[M-(H+NO_2_)]^+^ or[M-(CO+F)]^+^ or[M-(H+HCN+F)]^+^	61
Silylatedtemazepam	Y = 35.7× +0.064	0.977	0.500	0.714	343	[M-(H+CO)]+ or[M-(2H+HCN)]^+^	100
					283	[M-(OSi(CH_3_)_3_+H)]^+^	31
					257	[M-NCHOSi(CH_3_)_3_]^+^	36

a Limit of detection on column, IUPAC method.

b Limit of quantification on column, IUPAC method.

Calibration curves were also constructed for the drugs extracted from the drinks in a fashion analogous to those for pure compounds. The LOD and LOQ data are given in Table 4. The recovery of each of the drugs from each of the drinks was measured (Table 4) with values of diazepam 84–99%, flunitrazepam 56–101% and temazepam 81–105% depending upon the drink (Table 4). Recovery was least good from the white wine and flunitrazepam was particularly poor from the white wine (56%) and the Bacardi Breezer (77%). The reasons for this are more complicated than recovery merely being a function of the pH values of these drinks which lay in the range between pH 3.2 to pH 3.4 and the pK_a_ value of flunitrazepam (pK_a_ = 1.8) otherwise temazepam (pK_a_ = 1.6) should have been extracted equally poorly. The factors affecting recovery were not investigated further. Recovery of all drugs from both the beer and the vodka were good with mean values of 84–101%.

**Table 4:** Method validation and Kruskall-Wallis U test results from spiked beverages.

Spiked beverages		a ^,^ bLOD/ng	b ^,^ cLOQ/ng	dRecovery % ± eSD	fIs the difference significant at 95% CI?	
					RT storage	4°C storage
**Diazepam**	Bacardi Breezer	0.25	0.714	89±7.9	No	No
	Beer	0.571	1.43	84±8.4	No	No
	Vodka	0.286	1	99±2.9	No	No
	White wine	0.071	0.156	86±5.4	No	No
	J2O	0.143	0.5	86±6.5	Yes	No
**Flunitrazepam**	Bacardi Breezer	0.528	1.71	77±15	No	No
	Beer	1.07	1.5	101±20	No	Yes
	Vodka	0.356	1.14	97±4.1	No	Yes
	White wine	0.082	0.273	56±5.3	Yes	Yes
	J2O	0.643	2.11	85±6.5	Yes	Yes
**Silylated** **temazepam**	Bacardi Breezer	0.143	0.431	84±2.5	Yes	Yes
	Beer	0.071	0.357	97±13	Yes	No
	Vodka	0.143	0.357	90±6.7	Yes	Yes
	White wine	0.071	0.143	81±4.1	Yes	Yes
	J2O	0.214	0.693	105±10	Yes	Yes

a Limit of detection,

b IUPAC method,

c limit of quantification,

d n = 3,

e standard deviation,

f Kruskall-Wallis U test at 95% confidence interval.

### Stability of the Drugs under Different Storage Conditions

**Figure 1:**
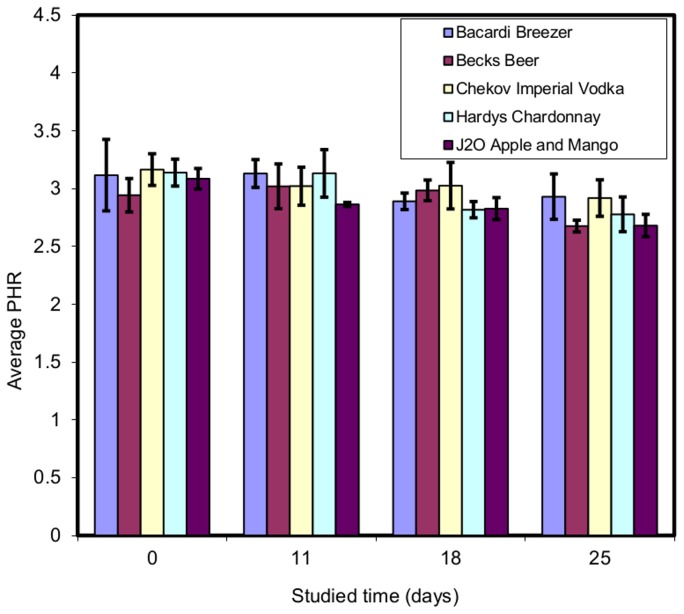
Peak height ratio (PHR) of diazepam: diazepam -d_5_ (n = 3).

**Figure 2:**
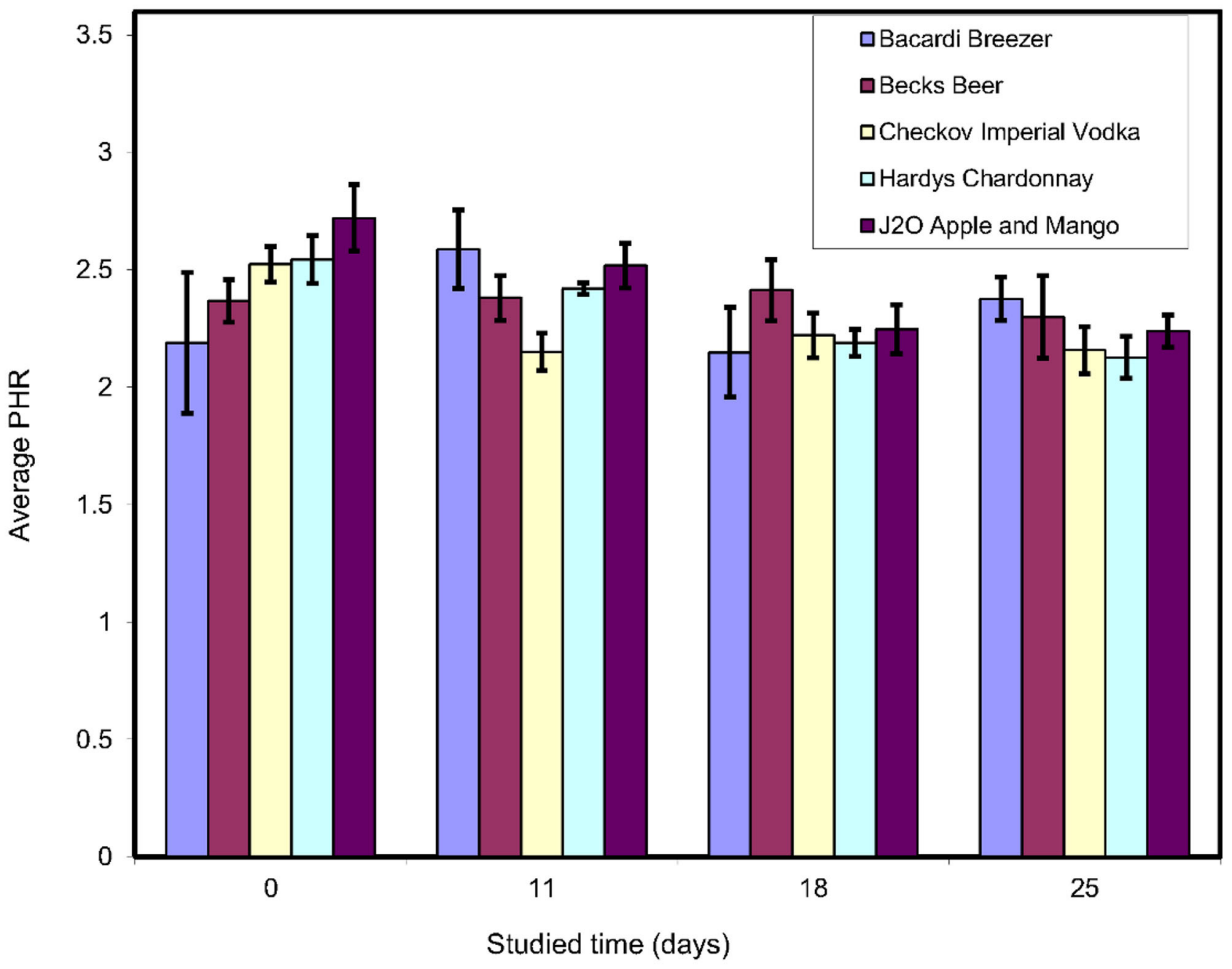
Peak height ratio (PHR) of flunitrazepam: diazepam -d_5_ (n = 3).

**Figure 3:**
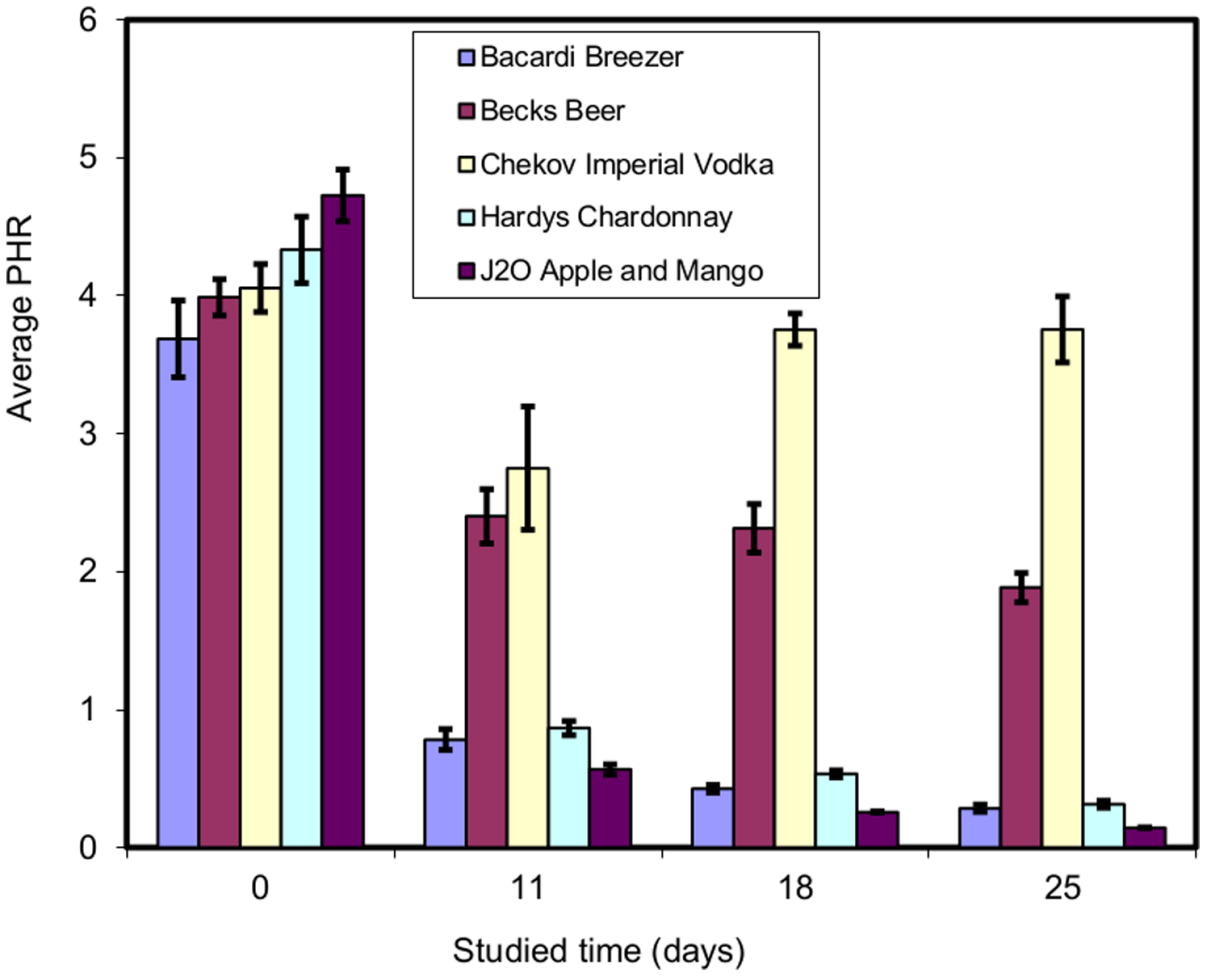
Peak height ratio (PHR) of silylated temazepam: diazepam -d_5_ (n = 3).

All of the spiked beverages stored at room temperature and at 4°C were subjected to LLE at the given time intervals. The results show that the drugs were detected under both storage conditions throughout the study period, although some drugs were more stable than others. Exemplar results for analytes extracted from drinks stored at room temperature for example, diazepam (Figure 1) flunitrazepam (Figure 2) and silylated temazepam (Figure 3) show a clear difference between the stability of the individual analytes in different beverages. In order to determine whether or not there is a significant difference in these results the Kruskal-Wallis U test was used. The chi-squared (χ2) statistic and p value were calculated and compared with the critical χ2 value at 95% confidence interval for 3 degrees of freedom and the summary of results are given in Table 4.

The Kruskal-Wallis U test showed that for diazepam there was no significant difference between the results obtained after storage at room temperature and at 4°C in any beverage except J2O (Figure 1, Table 4). In J2O, at room temperature, there was evidence of diazepam degradation. The cause of this was not investigated further. For flunitrazepam significant differences were observed after storage under both conditions in wine and J2O (Figure 2, Table 4). The instability is not linked to the sample pH and alcoholic content of the beverage because both of them have (i) same pH of 3.2 (ii) the wine is 12.5% v/v ethanol, the J2O is alcohol free. There was significant loss of temazepam in all the beverages analysed after storage in both conditions (Figure 3, Table 4) except beer at 4°C. In general the stability of benzodiazepines depended on the type of beverage in which they were dissolved. J2O in particular showed significant differences for all the analytes under both storage conditions.

Factors which might potentially impact upon the stability of benzodiazepines in a beverage are pH, alcohol concentration, extraction and analysis. Our results do not show any pattern related to them in the Kruskal-Wallis U significance tests.

### Simulated Case

Blind trial samples were extracted, derivatised and analysed following the method developed and presented in this paper. The drugs were each correctly identified. The concentrations of the drugs in the drinks lay between 95–100% of the true values. Against a true value of 0.083 mg/mL, the concentration of diazepam was detected at 0.079 mg/mL (95% recovery), 0.083 mg/mL (100% recovery) for temazepam from white wine.

## Conclusion

A GC-MS method for the simultaneous extraction and detection of three commonly detected benzodiazepines namely diazepam, flunitrazepam and temazepam has been adapted, validated and applied to beverages spiked with benzodiazepines implicated in DFSA cases. The advantage of this method is that it covers thermally stable and labile benzodiazepines. Thermal lability can be overcome through derivatisation using BSTFA-TCMS. The method presented here is selective, specific, yields good recovery of the analytes, provides linear calibration data and achieves lower LOD and LOQ than previously described.

The study of drugs in foodstuff and beverages is an important aspect in some forensic cases (HMA-v-Webster, 2012; R-v-Erin, 2012; R-v-Wilson) [26]–[28]. In these examples drugs were administered via foodstuffs and drinks and in the second of these cases, knowledge of the effect of prolonged dissolution of the drug would have been important for data interpretation. The same is true for benzodiazepines in drinks. Whilst benzodiazepines do not occur in beverages, it is important to know concentration in the drink, in some legislative systems, if interpretation of the effect that the drug would have is to be made.

There is a gap in the literature with respect to the stability of benzodiazepines in beverages over time and under different storage conditions. This study proves that diazepam, flunitrazepam and temazepam can be detected in Bacardi Breezer, beer, white wine, vodka and J2O for a 25 day period after storage at both room temperature and at 4°C. However, issues of stability exist for benzodiazepines in beverages, particularly for flunitrazepam and temazepam. This study demonstrates that such samples should be analysed as soon as possible after seizure regardless of how they are stored.

Additionally, due to the decomposition of the benzodiazepines it is not possible to determine the original concentration of the drug in the drink at the time of alleged administration. This has consequences in the forensic science context in that it may not be possible to determine whether a drug in a drink would have had an effect – especially when the drug concentration at the time of analysis is low. Of course, the presence of a drug in a drink should raise suspicion about the events that have led to its presence and association with the case under investigation. These spiked drinks will constitute additional evidence in DFSA casework.
